# Bionomics and distribution of malaria vectors in Kisumu city, Western Kenya: Implications for urban malaria transmission

**DOI:** 10.21203/rs.3.rs-4943539/v1

**Published:** 2024-09-19

**Authors:** Maxwell G. Machani, Shirley A. Onyango, Irene Nzioki, Sylvia Milanoi, Godfrey Nattoh, John Githure, Harrysone Atieli, Chloe Wang, Ming-chieh Lee, Goufa Zhou, Andrew Githeko, Yaw A. Afrane, Eric Ochomo, Guiyun Yan

**Affiliations:** Kenya Medical Research Institute; Kenyatta University; Kenya Medical Research Institute; Kenya Medical Research Institute; Kaimosi Friends University College; International Center of Excellence for Malaria Research, Tom Mboya University, Homa Bay, Kenya; International Center of Excellence for Malaria Research, Tom Mboya University, Homa Bay, Kenya; University of California, Irvine; University of California, Irvine; University of California, Irvine; Kenya Medical Research Institute; University of Ghana Medical School, College of Health Sciences, University of Ghana, Ghana; Kenya Medical Research Institute; University of California, Irvine

**Keywords:** Anopheles, malaria, Anopheles density, species composition, sporozoite infection, urban city

## Abstract

**Background:**

Increasing urbanization in tropical Africa may create new niches for malaria vectors, potentially leading to higher disease transmission rates. Vector control efforts remain largely targeted at ecologically rural bio-complexities with multiple hosts. Understanding mosquito species composition, ecology, host diversity and biting behavior in urban areas is crucial for planning effective control. This study assessed mosquito species diversity, abundance, behavioral patterns, and *Plasmodium* sporozoite infection rates of *Anopheles* vectors along an urban-rural transect in Kisumu city, western Kenya.

**Methods:**

Indoor and outdoor host-seeking and resting adult mosquitoes were collected using Centers for Disease Control and Prevention miniature light traps (CDC-LT) and mechanical aspirators (Prokopack) along an urban-rural transect. Females *Anopheles* mosquitoes collected were identified using morphological taxonomic keys to species level. Specimens belonging to the *Anopheles gambiae* complex and *Anopheles funestus* group were further processed using polymerase chain reaction (PCR) to identify members of each complex/group. Subsequently, sporozoite infection rates of the anopheline mosquitoes were determined using a multiplexed real-time quantitative PCR (qPCR) assay.

**Result:**

A total of 3,394 female *Anopheles* mosquitoes were collected and identified. These comprised of *An. gambiae* s.l. (68%), *An. funestus* group (19.8%), *An. coustani* (7.8%), *An. pharoensis* (2.6%), *An. maculipalipis* (1.6%), and *An. leesoni*(0.2%). All six species were found in urban zone, but only three were found in peri-urban and rural sites. Overall, urban collections accounted for the majority of these collections (55.5%) of mosquitoes collected, followed by those from peri-urban (30%) and rural sites (14.5%). Species distribution across the three ecotypes showed *Anopheles arabiensis* was the dominant species in urban (84.3%) and peri-urban (89%) sites, while *An. gambiae* s.s. was predominantly found in the rural zone (60.2%) alongside *An. arabiensis* (39.7%). *Anopheles funestus* was the predominant species in peri-urban (98.4%) and rural (85.7%) areas, with *An. leesoni* accounted for 1.6% and 14.3%, respectively. In urban areas, all samples from the *An. funestus* group were identified as *An. funestus* s.s.. Majority (55.5%) of *Anopheles* mosquitoes were collected indoors, while secondary vectors were primarily caught outdoors. Overall, sporozoite rates were higher outdoors 3.5% compared to indoors 1.45% in rural areas. Specifically, sporozoite infectivity rates for *An. funestus, An. gambiae* s.s and *An. arabiensis* collected indoors in the rural zone was 2.5%, 1.4% and 1% respectively. Outdoors in rural areas, *An. gambiae* had a sporozoite rate of 5.3%, while *An. arabiensis* had a rate of 2.1%. In peri-urban areas *An. gambiae* had a sporozoite rate of 2.3%. No sporozoites were detected in samples from urban sites.

**Conclusion:**

The study highlights a shift of diversity of *Anopheles* species towards urban areas with increased outdoor activity, and significant outdoor malaria transmission in rural and peri-urban areas, emphasizing the need for tools targeting outdoor-biting mosquitoes. The presence of *An. funestus* in urban settings is of interest and highlights the critical importance of sustained entomological surveillance to inform integrated vector control and prevent future transmission risks.

## Background

The sustained global malaria control campaign has made remarkable progress in reducing malaria morbidity and mortality, primarily by scaling up vector control tools and improving malaria case management (1). Nevertheless, recent findings from the World Health Organization (WHO) indicate that further reductions in malaria prevalence in Africa are not as significant, with progress stalling in several regions of sub-Saharan Africa where the disease remains widespread (2). The campaigns have predominantly focused on rural areas, overlooking urban centers where malaria prevalence has traditionally been low. However, malaria is now considered an emerging threat in rapidly urbanizing areas of sub-Saharan Africa (3, 4) highlighting the need to monitor vector populations and implement long-term interventions in the neglected urban environments. This oversight has gained significant attention, particularly with the recent establishment and spread of the invasive urban vector *Anopheles stephensi,* which is likely to alter disease risk landscape in Africa (5–7). In response to these challenges, the WHO introduced a framework supporting the control and elimination of malaria in urban environments, marking the beginning of efforts to address malaria in urban settings (8).

Urbanization, often associated with human development and progress, can also lead to significant inequalities and health problems (9, 10). The prevalence of *Anopheles* mosquitoes and malaria transmission in urban environments can be influenced by various factors, including housing conditions, land use patterns, population density, transportation/migration, and waste generation/pollution, among other anthropogenic practices (11–14). Peri-urban locations, which combine urban and rural characteristics, is likely to experience unique challenges due to changing environmental conditions and socioeconomic factors (15). Rural areas, with diverse ecological conditions and traditional practices, typically have higher mosquito densities and infection rates (16–18). However, many cities are now experiencing increased urban agriculture, poor drainage systems, broken and open sewers and inadequate housing due to rapid urbanization. These conditions create ideal environments for vector breeding and facilitate their entry into homes, significantly increasing the risk of exposure to malaria vectors (18–20). In addition, changing rainfall patterns may increase the availability and suitability of vector breeding habitats. Therefore, understanding mosquito-borne diseases in cities will require an integrative approach that combines ecological findings with their social context (21).

Although malaria vectors are uncommon in urban settings, they have adapted to human-induced changes, including climate change, which can potentially increase the risk of malaria transmission (22, 23). Over half the world’s population (4.2 billion people) now live in urban areas with the number expected to reach 9.7 billion by 2050. The proportion of urban residents in Africa is projected to increase from 36% in 2010 to 50% by 2030 and 60% by 2050 (24). In developing cities, large populations, particularly the poor, face significant challenges and often turn to activities like urban farming, which create favorable conditions for mosquitoes (25). High mobility from malaria-endemic rural areas and rural practices in urban regions, along with the recent presence of invasive species such as *Anopheles stephensi* (6, 26) and climate change (27, 28) in Kenya and other developing African cities, underscores the need for a robust mosquito surveillance in the urban centers. The main aim of this study was to assess malaria vector diversity, species composition, host-seeking and resting behaviors, and their contributions towards indoor and outdoor malaria transmission across urban, peri-urban and rural settings to provide critical insights for integrated malaria control strategies and targeted mitigation measures in urban areas.

## Method

### Description of Study Area

**Figure 1:**
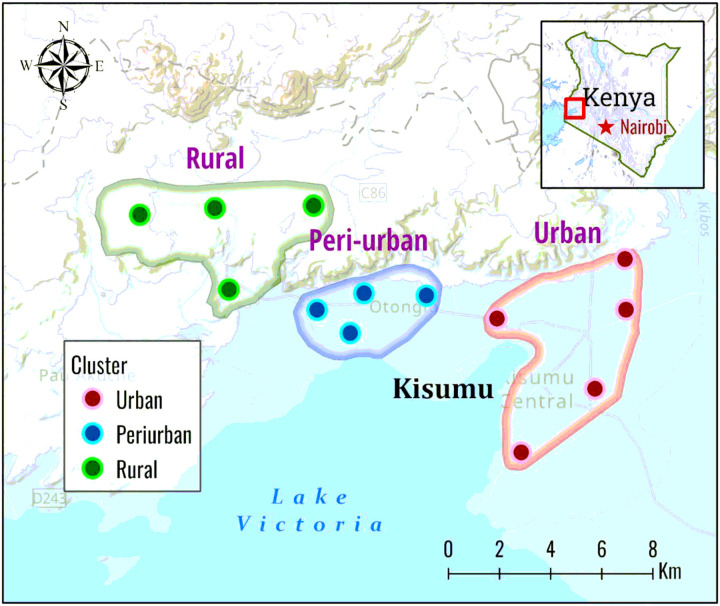
Map of Kenya (right corner) and Kisumu County (in expanded view) showing mosquito collection sites (circles) in the three sites (urban, peri-urban and rural areas in western Kenya

The study was conducted in an urban-rural continuum in Kisumu County in Western Kenya. Kisumu (00°06’S 034°45’E) is the third largest urban settlement in Kenya with a population of approximately 610,000 people and is located 10 km south of the equator on Lake Victoria. The city lies on the northeastern shore of Lake Victoria with an elevation of approximately 1,140 m above sea level. Kisumu city experiences a humid climate with an average relative humidity of 70%. Western Kenya has two distinct rainy seasons: a long rainy season from March to May and short rains in September through December. The extended dry season spans from January to March, with a shorter dry period from August to September. Annual rainfall typically ranges between 1,000 and 1,500 mm. Thirteen sites were randomly selected along an urban-rural transect from Kisumu city spanning a distance of 30 km. Among these, five locations: Nyalenda, Gesoko, Migosi, Mamboleo and Bandani were surveyed within the urban Kisumu and are characterized by dense urbanization. These sites all are informal residences located within the city). There were four sites in the peri-urban area: Kotetni, Kandalo, Tiengre and Kisian. The four rural locations sampled included Ojola, Mainga, Chulaimbo and Marera, which are approximately 30 km from the city (Fig. 1). Kisumu city is a major regional transportation hub where populations are engaged in formal and informal economic activity(18). *Anopheles* mosquito species in the peri-urban and rural of western Kenya lowlands include *Anopheles arabiensis, An. funestus,* and *An. gambiae* (29, 30).

#### Adult Anopheles collection

To determine the abundance of indoor and outdoor biting and resting adult female *Anopheles* mosquitoes in urban, peri-urban, and rural clusters, sampling was conducted from September 2022 to September 2023. CDC light traps (Model 512; John W. Hock Company, Gainesville, FL, USA) and mechanical aspirators (Prokopack) were used for these collections. For indoor and outdoor biting mosquitoes, battery-powered CDC light traps were hung at the foot end of the bed approximately 1.5 meters above the floor with a sleeping person protected under bednet indoors from 18:00 h to 06:00 h. and outdoors within 2 meters of sentinel houses (31). Collections were conducted in the morning from 6:00 h to 07:00 h. The trapping was done for four consecutive nights in five randomly selected houses in each cluster per zone. Prokopack aspirator (John W Hock, Gainesville, FL, USA) was used to collect indoor and outdoor resting mosquitoes from ten randomly selected houses every morning (06:00 h to 10:00 h) for four days in each cluster per zone. Indoor collections targeted mosquitoes resting on hunged clothes, walls, furniture, under roofs or ceilings, and under beds. Outdoor sampling included open containers, water reservoirs, outdoor kitchens, animal sheds, and outdoor human resting points. Each collection session in a house (both indoors and outdoors) lasted for approximately 20 minutes. Mosquitoes from each house and collection method were sorted, classified according to their gonotrophic status, and morphologically identified as *Anopheles* species following the recent taxonomic keys (32). Mosquitoes from each collection method were stored in vials labeled separately and preserved by desiccation. Different houses were visited throughout the study period.

### Identification of vector species complexes

A subset of members of *An. gambiae* s.l. and *An. funestus* s.l. groups randomly selected from indoor and outdoor collections from each cluster per zone were identified to species by polymerase chain reaction (PCR), following the protocols developed by Scott et al. for *An. gambiae* s.l. (33) and Koekemoer et al. for *An. funestus* s.l. (34).

### Molecular detection of Sporozoite infections

The head and thorax of the preserved *Anopheles* mosquito specimens were carefully separated from the abdomen, and DNA extracted from head/thorax using the alcohol precipitation method (35). The DNA was analyzed to determine sporozoite infections of *Plasmodium* species using a multiplexed real-time quantitative PCR (qPCR) assay. The assay was performed using the published species-specific 18 s ribosomal RNA probes and primers for *P. falciparum, P. malariae,* and *P*. *ovale* (36, 37).

### Data management and analysis

Vector densities from indoor and outdoor night collections were calculated as the number of female mosquitoes per trap/night for each collection method. Analysis of variance (ANOVA) was used to compare malaria vector density between indoor and outdoor locations. Differences in composition and abundance of mosquitoes between sites and locations were tested using chi-squared tests. The sporozoite rate was calculated as the proportion of *Anopheles* mosquito samples tested that turned positive for *Plasmodium* species. Data were stored in Microsoft Excel 2010 datasheets and analysis done using R statistical software (version 4.0.3; R foundation for Statistical Computing, Vienna, Austria).

## Result

### Mosquito species composition and abundance

During the study period, a total of 27,483 mosquitoes were collected, comprising 14,478 host-seeking and 13,005 resting mosquitoes, across urban, peri-urban, and rural sites. *Culex spp*. constituted the majority of the samples 87.6% (n = 24,089), while *Anopheles spp*. accounted for 12.4% (n = 3,394). The highest number of mosquitoes was collected in the urban zone, comprising 49.4% (n = 13,579) of the total captures. This was followed by the peri-urban zone with 37% (n = 10,164) and rural zone with 13.6% (n = 3,740) (Table 1).

### Anopheline mosquito species composition and abundance

Overall, a total of 3,394 adult female *Anopheles* mosquitoes, comprising six species, were collected over the study period. Of these, 55.5% (n = 1,883) were from the urban zone, 30% (n = 1,018) from the peri-urban zone, and 14.5% n = 493 from the rural zone (Table 1). The difference in the distribution of anopheline mosquito species between the study sites was statistically significant (F_2_, _1092_ = 14.45, P < 0.001). Overall, *Anopheles gambiae* s.l. was the predominant species, comprising 68% (n = 2,309) of the total collection. This was followed by *An. funestus* group (19.8%, n = 675), *An. coustani group* (7.8%, n = 263), *An. pretoriensis* (2.6%, n = 89), *An. maculipalpis* (1.6%, n = 53), and *An. pharoensis* (0.2%, n = 5). In the urban zone, *Anopheles gambiae* s.l was the most abundant 55.3% (n = 1042) followed by *An. funestus* group 25.5% (n = 480), *An. coustani* group 11.5% (n = 216), *An. pretoriensis* 4.7% (n = 89), *An. maculipalipis* 2.8% (n = 53) and *An. pharoensis* 0.2% (n = 3). Out of 1,018 *Anopheles* females collected in peri-urban, 88.2% (n = 898) were *An. gambiae* s.l, 8.4% (n = 86) *An. funestus* group and 3.3% (n = 34) *An. coustani* group. In rural zone, *An. gambiae* s.l was predominant species 74.8% (n = 396) followed by *An. funestus* group 22.1% (n = 109), *An. coustani* group 2.6% (n = 13) and *An. pharoensis* 0.4% (n = 2).

#### Indoor and outdoor Anopheles mosquito composition

**Figure 2:**
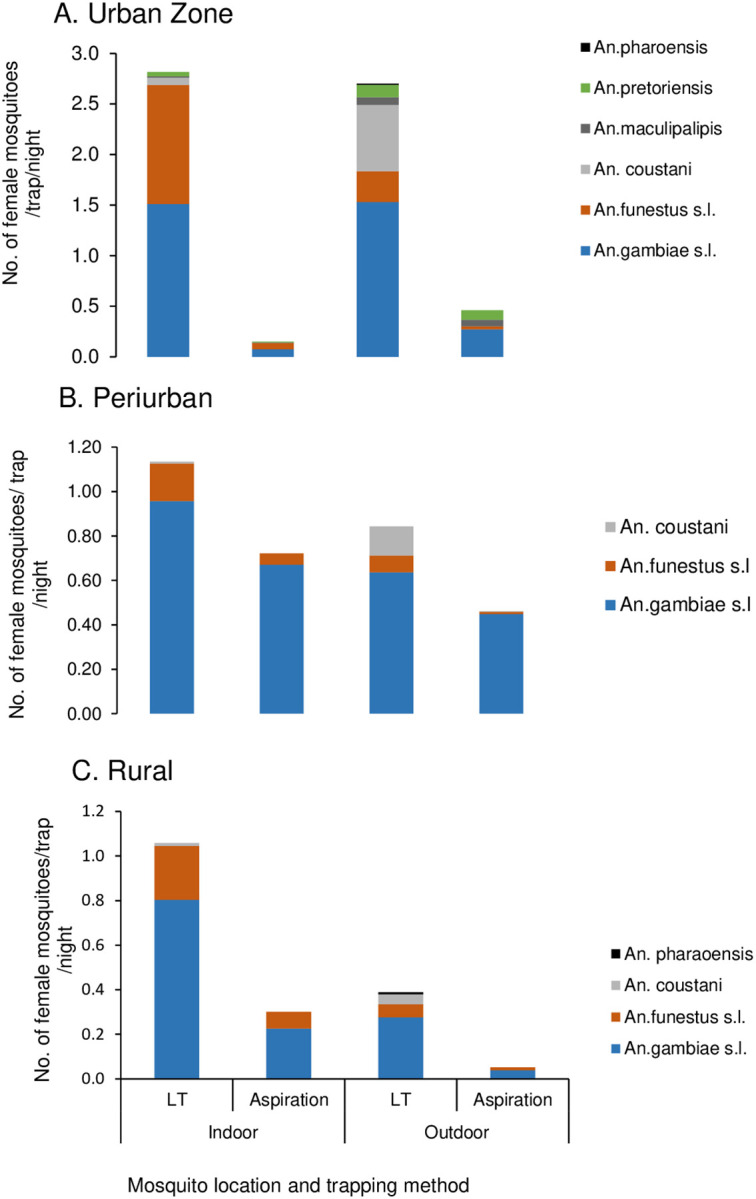
Indoor and outdoor resting density of female *Anopheles* mosquitoes collected per trapping method A: Urban and B: Peri-urban and C: Rural sites in Kisumu, western Kenya.

Overall, the majority of anophelines (55.5%, n = 1885) were collected indoors across the three zone. In urban, peri-urban, and rural sites, more *Anopheles* mosquitoes were host-seeking indoors [51.3% (95% CI 48.8–53.7%), 57.3% (95% CI 52.8–61.8%), and 73.1% (95% CI 68.3–78%), respectively] than outdoors [48.7% (95% CI 46.3–51.1%), 42.6% (95% CI 38.2–47.1%), and 26.8% (95% CI 22–31.8%), respectively]. The mean indoor host-seeking density for the *An. funestus* group in urban zone was significantly higher than the outdoor density (*t*_74_ = 2.67, p < 0.004) (Fig. 2A). In contrast, there was no significant difference in the mean indoor and outdoor host-seeking densities for *An. gambiae* s.l. in urban zone (p > 0.05). The secondary vectors mean outdoor host-seeking densities were marginally significant compared to the indoor densities for *An. maculipalipis* (t_9_ = 1.96, p < 0.04) and *An. coustani* group (*t*_42_ = 2.15, p<0 .02). The proportion of outdoor host seeking *An. pretoriensis* was higher 74% (95% CI 61.8–86.2%) compared to indoors 26% (95% CI 13.8–38.2%).

There was no significant difference in the mean indoor and outdoor host-seeking densities for *An. gambiae* s.l. and the *An. funestus* group (p > 0.05) in the peri-urban sites. The mean indoor host-seeking density for the *An. gambiae* s.l was significantly higher than the outdoor density (*t*_94_ = 2.3, p<0 .01), whereas the difference in the mean indoor and outdoor host-seeking densities for the An. *funestus* group was not significant (p > 0.05) in the rural zone. Most members of the *An. coustanigroup* were host-seeking outdoors in both peri-urban [94% (95% CI 85.7–100%)] and rural areas [76.9% (95% CI 54–99.8%)].

The majority of female *Anopheles* mosquitoes were caught resting outdoors [75.4% (95% CI 70–81%)] compared to indoors [24.6% (95% CI 19.1–30%)] in the urban zone. Conversely, in the peri-urban and rural sites, most female *Anopheles* mosquitoes were caught resting indoors [60.5% (95% CI 56.4–64.6%) and 85.2% (95% CI 79.8–90.5%), respectively] than outdoors [39.5% (95% CI 35.4–43.6%) and 14.8% (95% CI 9.4–20.1%), respectively]. The mean outdoor resting density of *An. gambiae* s.l in urban was significantly higher than indoor density (*t*_66_ = 2.2, p < 0.016) whereas, the difference in the mean indoor and outdoor resting density for *An. funestus* group was not significant (p > 0.05) (Fig. 2A). The majority of *An. maculipalipis* 93% (95% CI 82.7–100%) and *An. pretoriensis* 92.3% (95% CI 83.9–100.6%) were resting outdoors. The difference in mean indoor and outdoor resting densities for *An. gambiae* s.l and *An. funestus* group in peri-urban were not significant (p > 0.05) (Fig. 2B). In Rural, the mean indoor resting density of *An. gambiae* s.l was higher than outdoor *(t*_69_ = 1.76, p < 0.042) (Fig. 2C). The proportion of *An. funestus* group caught resting indoors 85.7% (95% CI 75.1–96.3%) was higher than outdoor 14.3% (95% CI 3.7–24.9%).

#### Anopheles gambiae and Anopheles funestus sibling species composition

**Figure 3:**
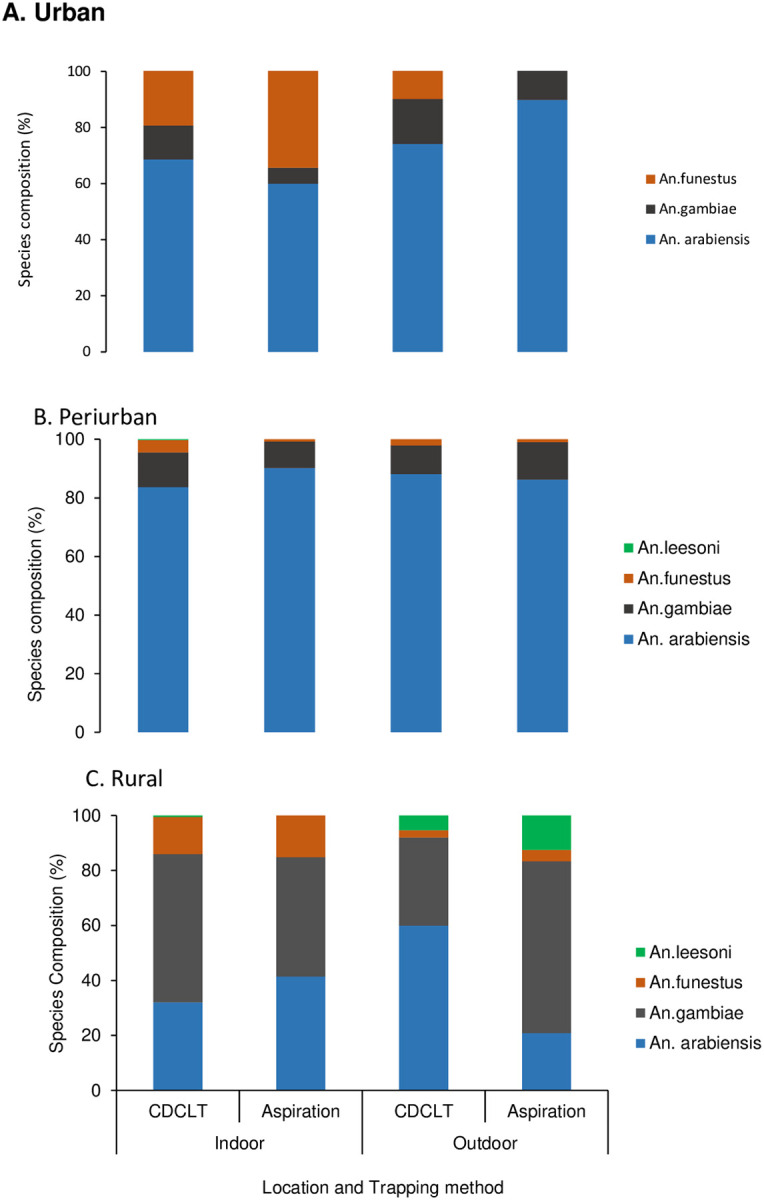
*Anopheles gambiaes*.l and *An. funestus* s.l sibling species composition, host-seeking and resting indoors and outdoors in A: urban and B: peri-urban and C: Rural sites Kisumu, western Kenya.

A total of 2,170 specimens (1,896 *An. gambiae* s.l. and 274 *An. funestus* group) were used for molecular assay to discriminate respective sibling species. *Anopheles arabiensis* was the predominant sibling species in both the urban (84.3%) and peri-urban (89%) sites, while *An. gambiae* accounted for 15.7% and 11% in these sites, respectively. In contrast, in the rural zone, *An. gambiae* s.s. (hereafter *An. gambiae)* was the most abundant species (60.2%), compared to *An. arabiensis* (39.7%). All the *An. funestus* group samples assayed from the urban zone were *An. funestus* s.s. (hereafter *An. funestus)* (Fig. 3A). In the peri-urban and rural sites, *An. funestus* was the dominant species (98.4% and 85.7%, respectively), while *An. leesoni* accounted for 1.6% and 14.3%, respectively (Fig. 3B&C). Overall, there was a significant difference between indoor and outdoor locations in terms of *An. funestus* group species composition (*X*^2^ = 21.34, *df=* 1, p< 0.001).

### Sporozoite infectivity rates

Sporozoite infectivity rate was used as a proxy for establishing *Plasmodium* infection rates. Out of the 2,170 mosquitoes tested, 8 specimens turned positive for sporozoites (i.e. 5 *An. gambiae,* 2 *An. arabiensis,* and 1 *An. funestus)*. Of these, one sample was from the peri-urban zone and seven from the rural zone. In the peri-urban zone, 2.3% (1/43) of *An. gambiae* collected outdoors tested positive for sporozoite. The Sporozoite rate for *An. gambiae* in the rural zone was 1.4% (2/139) indoors and 5.3% (2/38) outdoors. The sporozoite rate for *An. arabiensis* was 1% (1/99) indoors and 2.1% (1/48) outdoors. Additionally, 2.5% (1/40) of *An. funestus* collected indoors tested positive for sporozoites. Overall, the sporozoite rates were higher for samples collected outdoors 3.5% (3/86) than indoors 1.45% (4/278) in rural areas. None of the samples tested from urban zone were positive (Table 2).

## Discussion

With over half of the world’s population now residing in urban areas and projections suggesting this could rise to 75% by 2050 (38), rapid urbanization, often coupled with economic decline, has the potential to profoundly affect malaria epidemiology and control, thereby raising the disease burden in urban populations (9, 39). A major global public health concern is whether the rapid urbanization experienced in most developing African cities will shift malaria from rural to urban areas(40). Gaining insight into mosquito species composition, ecology, and biting behavior in these developing African cities is essential for implementing effective vector control strategies (41). This study found a surprisingly higher species diversity of anopheline mosquitoes in urban areas which was even higher compared to peri-urban and rural areas. The predominant vector was *An. gambiae* s.l. with *An. arabiensis* population being the highest in urban and peri-urban areas, while *An. gambiae* dominated the rural areas.

The higher numbers of *An. arabiensis* in the city corroborates similar studies in West Africa, which have demonstrated the increased adaptability of this species in urban environments (42, 43). This adaptability may be facilitated by urbanization-induced environmental changes, such as higher temperatures and lower humidity, which favor its survival (44). The abundance of *Anopheles gambiae* in rural areas can be attributed to its preference for unpolluted waters, which are commonly found in such settings. In contrast, urban environments, characterized by polluted waters, are less conducive to the survival of this species. However, instances of this species adapting to urban environments have been documented in Cameroon, West Africa (45). The presence of *An. funestus,* a significant malaria vector in rural sub-Saharan Africa, in urban areas is concerning as it could potentially sustain high levels of malaria transmission within cities. Reports of this species in urban areas of West Africa highlight their expansion to new niches thereby increasing the risk of malaria (45).

In addition to primary vectors, secondary vectors such as *An. coustani* group, *An. pretoriensis, An. maculipalipis* and *An. pharaonsis* were abundant in urban areas, unlike the rural and peri-urban settings where only the *An. coustani* group and *An. pharaonsis* were observed. A recent study from rural western Kenya reported an increase in secondary vectors (46) compared to previous findings (47). The co-occurrence of primary and secondary vectors in the urban zone is concerning as it may lead to increased risk of malaria transmission. Studies have shown that many secondary vectors prefer outdoor resting and biting, allowing them to sustain transmission even after indoor control measures, like insecticide-treated bed nets, have reduced primary vectors (48, 49). Some of the likely factors contributing to their occurrence in urban environments could be due to climate changes and unprecedented land-use contributing to their survival in urban environments (28, 48). The adaptation of the secondary vectors to the urban environment highlights the need for additional vector control interventions that target the behavior of these vectors, as well as a better understanding of their biology and role in urban malaria epidemiology to inform targeted interventions.

Consistent with previous studies in western Kenya (47, 50, 51), *An. arabiensis* was found to seek hosts and rest outdoors more frequently than indoors in urban and rural sites but showed no such preference in peri-urban areas. This variability may be influenced by ecological factors and implemented indoor vector control measures (52, 53), challenging the traditional indoor-focused interventions. Conversely, *An. funestus* and *An. gambiae* consistently exhibited indoor host-seeking and resting behaviors despite the use of LLINs, likely as a result of high insecticide resistance (51, 54). Moreover, these behaviors may also be influenced by poor housing conditions, which frequently fail to prevent mosquitoes from entering homes. While urbanization often improves infrastructure and housing quality, providing better mosquito-proof environments and healthcare access, this improvement may not extend to many developing African cities with slum-like conditions, as observed in this study. Thus, effective vector control strategies like house screening and larval source management are necessary to mitigate mosquito entry and outdoor mosquito activities in such settings. Secondary vectors like *An. maculipalipis, An. coustani, An. pretoriensis,* and *An. pharaoensis* showed increased outdoor activity, particularly in urban areas, potentially evading primary interventions and sustaining malaria transmission. Their ability to harbor *Plasmodium* parasites(46, 55) emphasizes their significant epidemiological impact, highlighting the need for robust entomological surveillance and targeted vector control strategies.

Malaria persistence is linked to behavioral changes in anopheline mosquitoes (56). This study found that most malaria transmission by *An. funestus* likely occurs indoors in rural areas, confirming its significant role in indoor transmission. Conversely, *An. arabiensis* and *An. gambiae* may be more involved in outdoor transmission, with *An. gambiae* potentially driving outdoor malaria transmission in peri-urban areas. Overall, most transmission occurred outdoors in rural and peri-urban areas, suggesting that indoor vector control methods like LLINs and IRS alone may not be sufficient, as outdoor-biting vectors pose a significant threat to elimination efforts. Despite the high numbers of malaria vectors in urban areas, which could suggest ongoing malaria transmission, no *Plasmodium* infections were detected in the tested mosquitoes. This absence of detection could be due to the limitations of the CDC LT trap in high-light urban environments. In western Kenya, CDC light traps in areas dominated by *An. arabiensis* have been found to capture a higher proportion of younger mosquitoes, confirmed by parity dissections(57). Additionally, urban-adapted malaria vectors have been reported to have a shorter lifespan compared to rural counterparts (4.1 days versus 11 days) (58), potentially limiting their ability to transmit the parasite. Although this parity and survivorship information were not considered in the current analysis, integrating these factors in future research could enhance our understanding of mosquito population dynamics in urban areas. Nonetheless, studies in West Africa have implicated *An. arabiensis* and *An. funestus* to urban malaria transmission, necessitating the need for tailored urban-specific vector control strategies. It is concerning that secondary vectors such as *An. coustani, An. pretoriensis,* and *An. pharoensis,* despite their tendency to feed on animals, have been found susceptible to *Plasmodium* infections (46, 47, 55). The complex behaviors and species diversity of these vectors in urban areas, pose a significant challenge to malaria elimination efforts that rely solely on indoor vector control, underscoring the need for ongoing adoption of integrated control strategies.

## Conclusion

The study revealed a high diversity of *Anopheles* species in urban areas, with significant outdoor activity. The detection of *An. funestus* in urban environments is concerning due to its established role in malaria transmission in rural areas, where malaria is high. Notably, outdoor malaria transmission was prevalent in rural and peri-urban regions, emphasizing the need to adapt and diversify interventions targeting outdoor-biting and resting mosquitoes. These findings highlight the importance of increased routine entomological surveillance in urban areas. Implementing integrated vector control measures, including larval source management, house modifications such as screening windows and eaves, and improved urban planning, is crucial for effective urban vector control.

## Figures and Tables

**Table 1:** Morphologically identified adult mosquitoes samples by zones (urban, peri-urban and rural) based on sampling method and location in Kisumu city.

Zone	Mosquito species	Indoor			Outdoor			Total
		LT	Aspiration	Total	LT	Aspiration	Total	
Urban	*An.gambiae* s.l.	453	29	482	455	105	560	1042
	*An.funestus* grp	354	24	378	90	12	102	480
	*An. coustani* grp	21	0	21	195	0	195	216
	*An.maculipalipis*	4	2	6	22	25	47	53
	*An.pretoriensis*	13	3	16	37	36	73	89
	*An.pharoensis*	0	0	0	3	0	3	3
	*Total Anopheles*	845	58	903	802	178	980	1883
	*Culex spp*	3663	2182	5845	4389	1462	5851	11696
Peri-urban	*An.gambiae s.l*	227	309	536	151	211	362	898
	*An.funestus* grp	40	23	63	18	5	23	86
	*An. coustani* grp	2	0	2	31	1	32	34
	*Total Anopheles*	269	332	601	200	217	417	1018
	*Culex spp*	2021	3339	5360	1748	2038	3786	9146
Rural	*An.gambiae* s.l.	180	108	288	62	19	81	369
	*An.funestus* grp	54	36	90	13	6	19	109
	*An. coustani* grp	3	0	3	10	0	10	13
	*An. pharaoensis*	0	0	0	2	0	2	2
	Total *Anopheles*	237	144	381	87	25	112	493
	*Culex spp*	740	648	1388	470	1389	1859	3247

**Table 2:** Sporozoite rates of *Anopheles* mosquitoes from indoor and outdoor collections in Urban, peri-urban and rural zones in Kisumu, western Kenya

Study zone and *Anopheles* species	Parameters	Indoor			Outdoor			Overall
		LT	Aspiration	Total	LT	Aspiration	Total	
Urban								
*An.gambiae* s.s	No.tested	39	2	41	41	10	51	92
	Pf + Ve (%)	0	0	0	0	0	0	0
*An.arabiensis*	No.tested	221	21	242	189	80	269	511
	Pf + Ve (%)	0	0	0	0	0	0	0
*An.funestus s.s*.	No.tested	76	15	91	27	0	27	118
	Pf + Ve (%)	0	0	0	0	0	0	0
*An. coustani*	No.tested	3	0	3	48	0	48	51
	Pf + Ve (%)	0	0	0	0	0	0	0
*An.ziemanni*	No.tested	0	0	0	26	0	26	26
	Pf + Ve (%)	0	0	0	0	0	0	0
*An. maculipalipis*	No.tested	1	0	1	22	15	37	38
	Pf + Ve (%)	0	0	0	0	0	0	0
*An.pretoriensis*	No.tested	8	0	8	35	31	66	74
	Pf + Ve (%)	0	0	0	0	0	0	0
Peri-urban								
*An.gambiae* s.s	No.tested	35	18	53	20	23	43	96
	Pf + Ve (%)	0	0	0	0	1(4.4)	1(2.3)	1(1.0)
*An.arabiensis*	No.tested	223	188	411	152	165	317	728
	Pf + Ve (%)	0	0	0	0	0	0	0
*An.funestus* s.s.	No.tested	8	2	10	3	2	5	15
	Pf + Ve (%)	0	0	0	0	0	0	0
*An. coustani*	No.tested	0	0	0	21	0	21	21
	Pf + Ve (%)	0	0	0	0	0	0	0
Rural								
*An.gambiae* s.s	No.tested	96	43	139	23	15	38	177
	Pf + Ve (%)	0	2(4.7)	2(1.4)	0	2(13.3)	2(5.3)	4(2.3)
*An.arabiensis*	No.tested	58	41	99	43	5	48	147
	Pf + Ve (%)	1(1.7)	0	1(1.0)	0	1(20)	1(2.1)	2(1.4)
*An.funestus* s.s.	No.tested	25	15	40	1	1	2	42
	Pf + Ve (%)	0	1(6.7)	1(2.5)	0	0	0	1(2.4)
*An.leesoni*	No.tested	1	0	1	4	3	7	8
	Pf + Ve (%)	0	0	0	0	0	0	0

## Data Availability

The dataset supporting the conclusions of this article is included within the article and its supplementary information files.
